# Investigation on acoustic emission characteristics of fault stick-slip under different lateral pressures

**DOI:** 10.1038/s41598-024-57076-0

**Published:** 2024-03-20

**Authors:** Ling Ding, Yangfeng Zhao, Yishan Pan, Yi Fan

**Affiliations:** 1 School of Mechanics and Engineering, Liaoning Technical University, Fuxin, 123000 Liaoning China; 2 Liaoning Key Laboratory of Mining Environment and Disaster Mechanics, Liaoning Technical University, Fuxin, 123000 China; 3 Institute of Disaster Rock Mechanics, Liaoning University, Shenyang, 110036 Liaoning China

**Keywords:** Fault stick-slip, Acoustic emission, Fractal characteristics, Lateral pressure, Natural hazards, Physics

## Abstract

To explore the effect of different stress environments on fault-slip rockbursts. Bidirectional shear friction experiments with different lateral pressures were conducted on precracked syenogranites buried at 800 m. The macroscopic statistical parameters (cumulative number of AE events, magnitude and *b value*) and local characteristic parameters (amplitude and dominant frequency) of acoustic emission during the stick-slip process under different lateral pressures were investigated. In addition, based on fractal theory, the nonlinear characteristics of AE spectrum were analyzed. On this basis, the microscopic mechanism of fault stick-slip was discussed. The results show that the lateral pressure influences the friction strength of the fault and stick-slip motion characteristics. With increasing lateral pressure, the proportion of transgranular shear fractures increases, which leads to an increase of cumulative number of AE events and magnitude. The periodic decrease in the *b* value is more significant at high lateral pressure. There is a good correlation between a high-magnitude AE event and a stress drop. The AE frequency with phased response characteristics can be used to effectively identify the evolution of fault stick-slip instability at the laboratory scale. A sharp increase in the amplitude of the dominant frequency can be regarded as one of the precursory features of fault stick-slip instability. The AE frequency spectra have multifractal characteristics, that differ among the different stages. The maximum multifractal dimension and spectral width can reflect the difference in energy released during fault stick-slip motion.

## Introduction

In recent years, the number of deep rock mass engineering projects, such as deep mining and deep tunnels, has increased, and the frequency, intensity and damage degree of fault-slip rockbursts have risen linearly due to the high crustal stress environment. Therefore, accurate and timely prediction is highly important for preventing rockbursts from occurring. Acoustic emission signals contain a large amount of core information related to the process of rock fracture evolution, and are widely used in monitoring and preventing dynamic disasters in coal and rock because of the advantages of directional, noncontact and continuous monitoring^1^. Fault stick-slip motion is the mechanism of fault-slip rockburst^2^. Studying the influence of different stress environments on fault stick-slip evolution and AE response characteristics is highly important for deep engineering dynamic disaster prediction.

Domestic and foreign scholars have conducted various theoretical studies and experimental analyses on fault stick-slip motion. Shi et al.^3^ established a model of fault instability due to inhomogeneous and progressive weakening of the fault, and divided the fault plane into slipped and locked regions. Mi et al.^4^ studied the effect of geometrically irregular bodies on the mechanical behavior of fault activity and reported that a fault zone containing extensional jogs was characterized by velocity weakening and could be described by a rate and state friction law. Zhou et al.^5^ found through simulation that the stress distribution along a simulated fault was highly nonuniform, which dictated the location at which stick-slip failure initiated. Nucleation started at a first-yield material point, and as the energy accumulated in the heterogeneous fault, a nucleation zone progressively formed. Chen et al.^6^ attributed the sticking phase to the locking of touching asperities and the slipping phase to the brittle failure of these asperities, and found that the fault asperities were as strong as the inherent strength of the host rock. Zhao et al.^7^ applied the shear beam model for interface failure to analyze fault rockburst occurrence, and obtained the analytical relationship between various geometric and mechanical parameters during fault rockburst. Chen et al.^8^ established a self-locking model of rock friction and reported that an increase in confining pressure contributed to the occurrence of stick-slip motion. Mu et al.^9^ reported that as the confining pressure increased, the fault zone transitioned from unstable sliding to stable sliding. Cui et al.^10^ studied the energy dissipation and release of fault gouge, and reported that with increasing cell pressure, the deviatoric stress drop increased, and the ratio of the deviatoric stress drop to the maximum deviatoric stress gradually stabilized. Paglialunga et al.^11^ conducted triaxial compression tests on granite samples under crustal conditions (15–120 MPa) and reported that coseismic changes were mostly controlled by the elastic reopening of microcracks in the bulk rather than by coseismic damage or the formation of fault gouge. Cui et al.^12^ conducted friction experiments on precracked granodiorite specimens, and reported that lateral pressure disturbance had a more significant effect on fault stick-slip. Song et al.^13^ reported that the peak stress and stress drop increase during fault rockburst when the confining pressure increases.

Due to the presence of asperities in the fault plane, the distribution of fault strength is not uniform, and microfractures occur locally before the fault slips^14^. An AE signal can be observed^15^. Through the AE signal, it is possible to infer the internal behavior changes of rock and invert the failure mechanism^16^. Many studies have focused on the AE characteristics of fault stick-slip motion. Zhou et al.^17^ conducted precursor prediction of laboratory earthquake timing in granite samples and determined that the most effective AE parameter for forecasting sliding instability time was the AE event, followed by the amplitude, ringing count and rise time (RT). Hou et al.^18^ presented an AE monitoring method based on the matched filter technique. Song et al.^19^ studied the AE characteristics of deformation evolution during frictional sliding, in rocks. The corresponding relationships between the interface frictional sliding rate and the AE ringing count, the interface frictional sliding displacement and the AE cumulative energy, the specimen deformation energy density and the AE *b* value were analyzed. Li et al.^20^ developed an ultrahigh-speed, multichannel and continuous recording data acquisition system for deformation measurements and reported that coseismic processes could include multiple rupture events and that each event had its own AE waveform distinguishable in time. Goebel et al.^21^ investigated variations in the seismic *b* value of AE events during stress buildup and release in laboratory-created fault zones. The *b* values mirror periodic stress changes that occur during a series of stick-slip events, and are correlated with stress during many seismic cycles. Liu et al.^22^ suggested that according to the temporal sequence of occurrence of different types of AE, the AE rate, *b* value and local failure signal could be applied to predict or early predict geological hazards. Thompson et al.^23^ reported that asperity shear caused fault stick-slip instability, and damage to the fracture zone led to a decrease in the *b* value and fractal dimension. Xie et al.^24^ introduced fractal theory and the multifractal detrended fluctuation analysis (MF-DFA) method to estimate the waveform multifractal spectrum of goaf rock AE signals and investigated multifractal time-varying response characteristics. Zhang et al.^25^ and Kong et al.^26–28^ found that the multifractal spectrum can explain AE energy signals frequency responses and the causes of AE events with load, multifractal spectrum width can reflect the differences between the large and small AE energy signals, and another parameter (*∆f*) can reflect the relationship between the frequency of the least and greatest signals in the AE energy time series. Niu et al.^29,30^ used multifractal theory to study the deformation damage process of single-flawed red sandstone with different wavilness angles and shale specimens, established a quantitative criterion based on multifractal parameters of AE time series, and considered that the combined application of multifractal parameters can be regarded as a precursor in the deformation and fracture damage process of flawed rocks. Qiu et al.^31^ used Hilbert-H and multifractal theory to study the refined nonlinear characteristics of EMR and AE during coal splitting failure, revealing valuable information pertaining to coal fracture law contained in EMR and AE waveform. Liu et al.^32^ analyzed the dynamic multifractal characteristics of AE of composite samples and found that the multifractal spectrum of coal AE energy, rock AE energy and count in the combined samples is right hook-shaped, and the multifractal spectrum of coal AE count is a bell-shaped curve.

The basic feature of the stress state of a deep rock mass is that the geostress gradually changes to a state of deep hydrostatic pressure with increasing depth^33^. Simulating the deep pressure environment in the laboratory and monitoring the AE characteristics of rock deformation and fractures are effective methods for predicting deep engineering dynamic disasters. Jiang et al.^34^ used Pb as the confining pressure medium to conduct triaxial friction experiments on granite, and reported that the occurrence of AE events advanced with increasing confining pressure. Hao et al.^35^ analyzed the effects of confining pressure on the mechanical properties and AE characteristics of coal reservoirs under high confining pressure. Zhang et al.^36^ reported that under lower confining pressures, the *b* value of AE signals before rock failure decreased dramatically, indicating that brittle failure of the rock occurred. Ji et al.^37^ conducted laboratory experiments on the characteristics of AE in two frequency channels during the full failure process of granite specimens under different confining pressures, and found that the number of concentrated ranges of peak frequencies of AE increased before major cracking of the rock. Dong et al.^38^ observed that the *b* values exhibited a decreasing trend before fracture and tended to decrease with increasing confining pressure. Thus, the *b* value can be used as an indicator for validating the stress concentration area, including the magnitude and accumulative probability density distribution of events, which is a beneficial complement to clarifying precursor information of rock mass instability.

Although there are many studies on fault stick-slip motion, most of them have focused on the AE response during the meta-instability stage, and have rarely involved whole-process AE characteristic analysis. The existing precursory characteristics of fault stick-slip instability are not sufficient to meet early warning requirements. The single warning parameter has limitations, as it cannot be used to effectively identify precursors of rock disasters in practical applications and may cause false alarms and missed alarms. In this study, to solve the abovementioned problems, bidirectional shear friction experiments under different lateral pressures were conducted on precracked syenogranites buried at approximately 800 m, and the mechanical behaviour of fault stick-slip was observed. The macroscopic statistical parameters (AE event, magnitude and *b value*) and local characteristic parameters (AE amplitude and dominant frequency) during the stick-slip process are studied. In addition, based on fractal theory, the nonlinear characteristics of AE spectrum are analyzed. On this basis, the microscopic mechanism of fault stick-slip motion is discussed. These research results are highly important for further improving the automatic identification efficiency of AE waveforms, monitoring and early warning systems for fault stick-slip instability, and investigating the mechanism of fault AE generation.

## Experimental methods

### Specimen preparation

**Figure 1:**
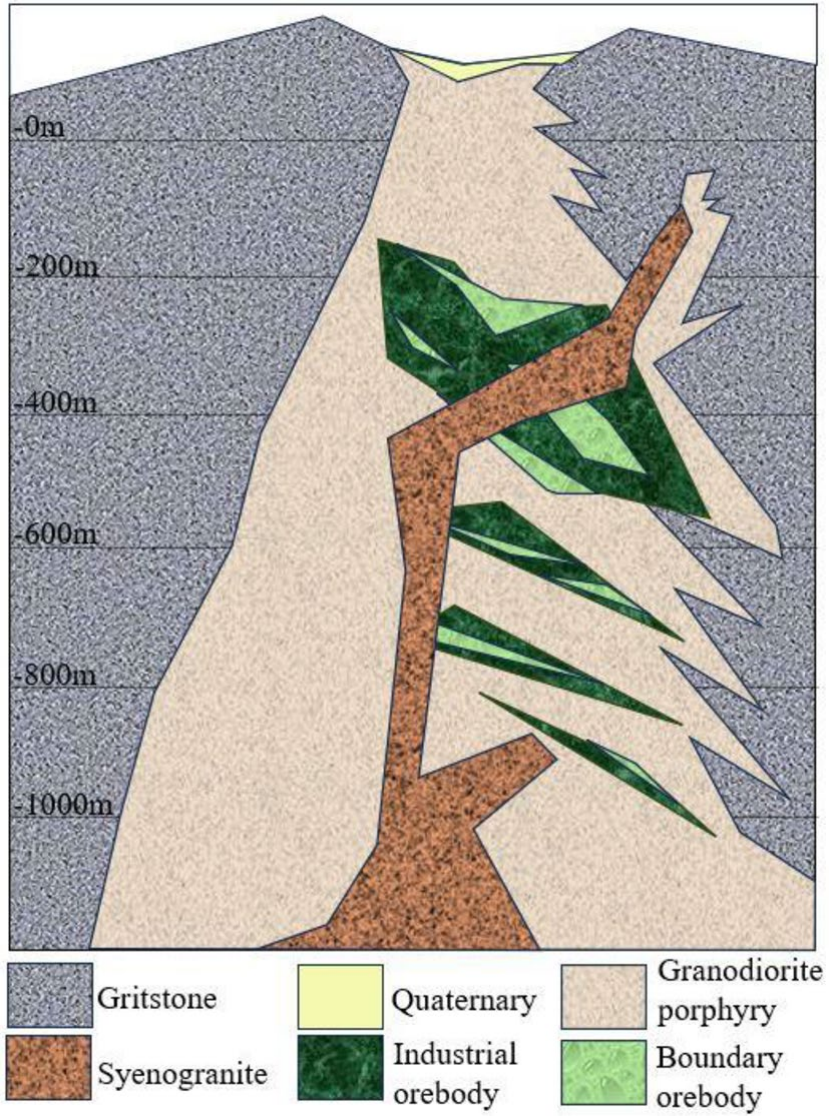
Simplified geological map sampling location.

The syenogranite used in the experiment was taken from Tongling Mine concentration area, and the geological map of sampling location map is shown in Fig. 1. Tongling area is one of the important non-ferrous metal bases in China. There are six mineral fields distributed in the area of about 1000 km^2^, among which the main shaft of Dongguashan Copper Mine is the deepest non-ferrous metal mine in China, with a digging depth of 1125 m. The multi-level, multi-direction and multilevel fault between basement and cap layer in Tongling mine area lead to frequent high-magnitude dynamic disasters. Nine syenogranite specimens of the same size with areas of 300 mm by 200 mm and thicknesses of 25 mm were used in the experiments. The uniaxial compressive strength, Poisson’s ratio and shear modulus of the specimens are 164 MPa, 0.28 GPa and 26.7 GPa, respectively. The specimens were cut diagonally to form macroscopic planar faults with a fault dip of 56°. The fault planes of the specimens were ground by a 150-mesh (with an abrasive grain size of 110 μm) RVD (Resinoid and Vitrified bonded Diamond grains) abrasive before the experiments were conducted.

### Experimental system

**Figure 2:**
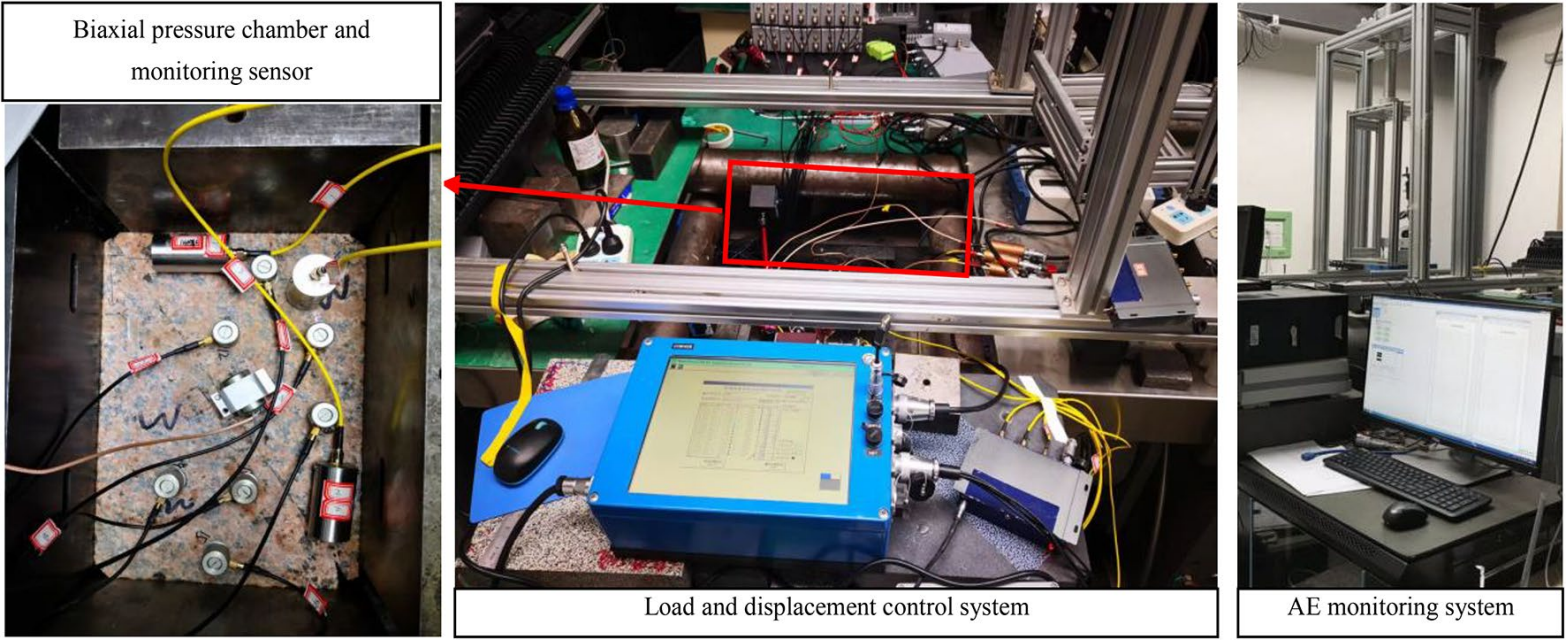
Experimental system.

The experimental system is shown in Fig. 2, including a loading system and an AE signal acquisition system. The loading control system adopts the horizontal biaxial hydraulic servo control loading machine developed by the Institute of Geology of China Earthquake Administration, which can realize the bidirectional control of displacement or load. The bidirectional load range is 0–150 t, the load control rate range is 10–1000 kN/s, and the displacement control rate range is 0. 01–700 μm/s.

**Figure 3:**
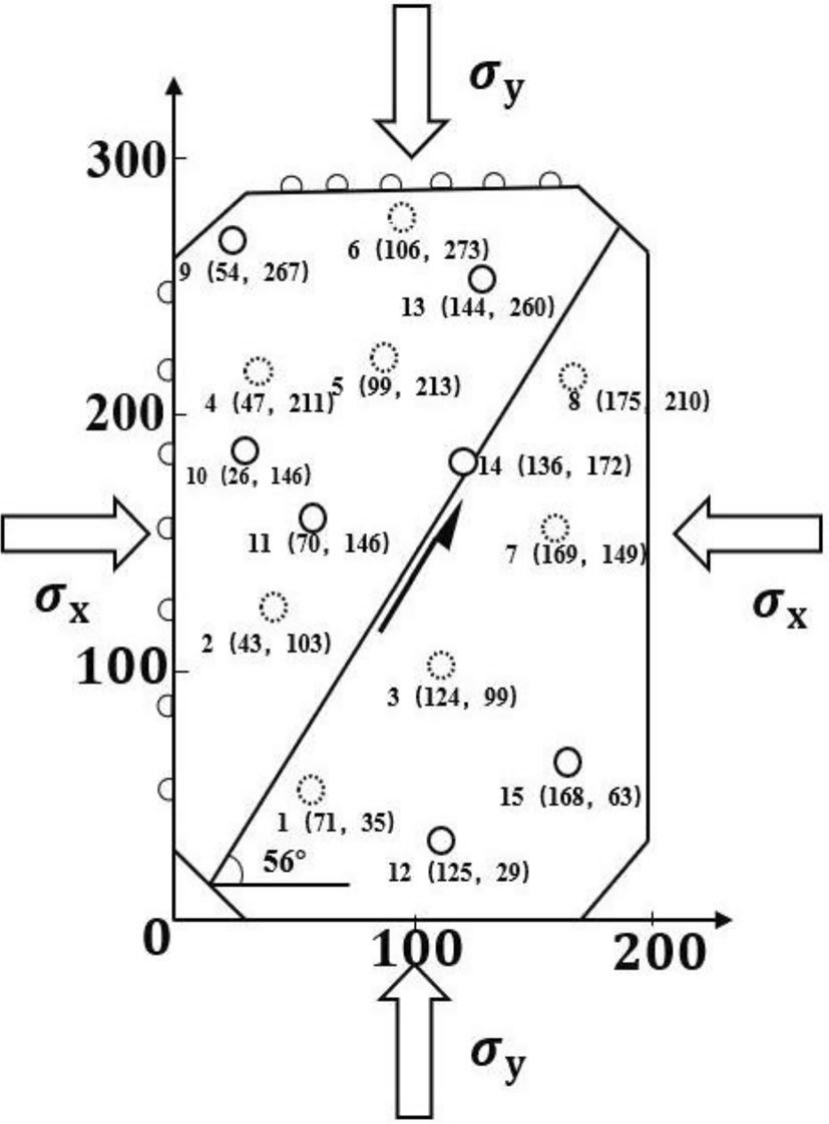
Specimen structure and AE sensor location.

The AE data acquisition system has a 16 -channel full waveform recorder, a sampling frequency of 3 Msps, a preamplifier of 40 dB, and a sampling length of 4096 specimen points. The AD conversion resolution of the AE instrument is 16 bits. AE sensors were dispersed on the surface of the specimen, and coupling agents were used to reduce the attenuation of AE signals. The sensor arrangement is shown in Fig. 3. Circles 1–15 in the figure are the positions of the AE sensors, among which sensors 1–8 are on the upper surface of the specimen; and sensors 9–15 are on the lower surface of the specimen. It is worth emphasizing that the experimental system can realize nanosecond synchronization of the force signal and AE signal of different channels. During loading, the AE signal was collected and stored, and the threshold value of signal acquisition was raised to eliminate the external interference signal.

### Experimental procedure

Li et al.^39^ sampled the geostress suffered by the coal in many places and obtained the fitting formula for calculating geostress. According to this, the approximate geostress values of syenogranite with different burial depths are obtained, and the initial geostress loading values of the experiment are determined. The specific loading scheme is shown in Table 1. During the experiments, the specimens were placed in a horizontal biaxial hydraulic servo control loading apparatus. Before the experiment started, the time synchronization between the load system and the AE signal acquisition system was set. To ensure that the data of each system could be strictly corresponding in time. Taking experiment A1 as an example, the loading process was as follows: first, the load in the X and Y directions was synchronously applied to 5 MPa at a speed of 50 kg/s by controlling the loading pattern, and then the X direction was kept at the 5 MPa level. The Y direction was transferred to the controlling pattern of paten displacement, and the pressure was continued at a loading rate of 1 um/s until regular stick-slip events occurred. The data of several stable stick-slip events were recorded under different lateral pressures. There are 3 parallel specimens in the same group. Since the stress and AE signal characteristics of the same group are similar, 3 typical specimens are selected for analysis and research.

**Table 1:** Test scheme parameters.

Buried depth/(m)	Actual geostress/MPa			Experimental number	Size/(mm×mm×mm)	Dip angle/(°)	Experimental applying lateral pressure/(MPa)	Loading rate/(μm s−^1^)
	Vertical stress	Maximum horizontal stress	Minimum horizontal stress					
140	5.107	10.98	3.524	A1	300 × 200 × 25	56	5	1
				B1				
				C1				
420	10.931	17.644	8.676	A2			15	
				B2				
				C2				
840	19.667	27.64	16.404	A3			25	
				B3				
				C3				

### Stress characteristics of stick-slip

**Figure 4:**
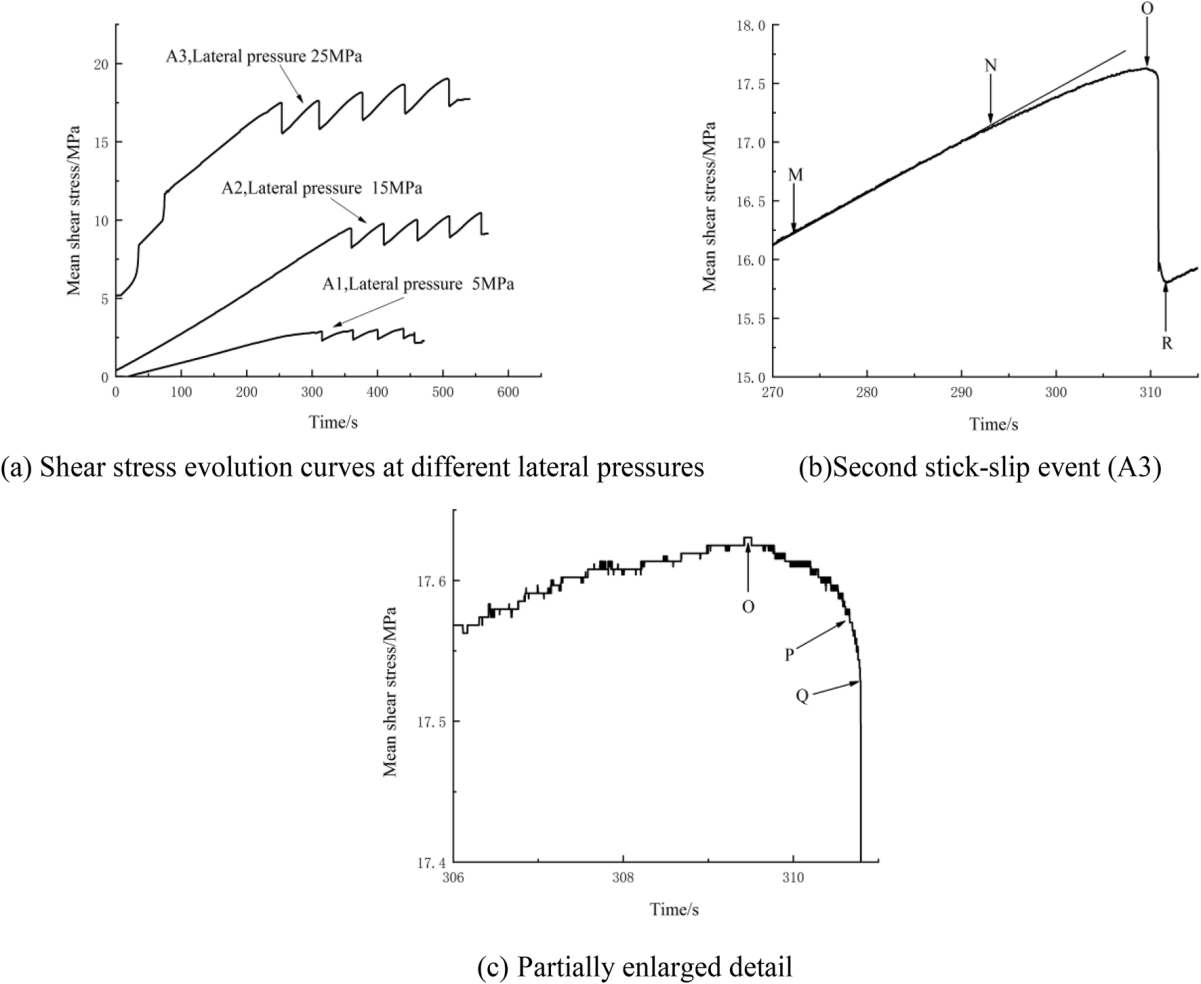
Shear stress evolution curves.

In the data analysis, the mean shear stress was calculated from the stresses along the diagonal direction of the specimen. The shear stress curves are shown in Fig. 4a, the shear stress of the second stick-slip event is shown in Fig. 4b, and the partial enlarged detail of the shear stress is shown in Fig. 4c.

Figure 4a indicates that the variation trends of the stick-slip curves under the different lateral pressures are approximately the same. At the initial stage of loading, the mean shear stress increases, and when the shear stress accumulates to the frictional strength, fault slip dislocation occurs. Once stick-slip occurs on the fault, it occurs again, accompanied by a change in fault displacement. To make the fault slip again after each slip, the shear stress on the fault plane must increase. To avoid interference caused by excessive fault displacement, only the first 5 stick-slip events are selected in this paper. A complete stick-slip event is shown in Fig. 4b,c. According to the evolutionary trend of shear stress, the key time points during fault deformation are marked as M, N, O, P, Q, and R. Specifically, M-O represents the shear stress build-up stage, O-Q- represents the meta-instability stage, and Q-R- represents the stick-slip instability stage. The shear stress of M–O does not change linearly, but increases in a stepwise way. At the shear stress build-up stage, the fault plane resistance gradually homogenizes. From point N, the stress accumulation flattens, and the distribution of resistance approaches zero. The shear stress corresponding to point O is the peak stress, and the meta-instability stage includes two parts: O-P-quasi-static stress initiation, and P-Q-quasi-dynamic stress release. At the meta-instability stage, the fault changes from energy accumulation to energy release, and the meta-instability stage is the final stage before fault instability^40^. At the stick-slip instability stage, the shear stress decreases sharply, and energy is released in the form of strain.

In Fig. 4a, for the A1 fault with 5 MPa of lateral pressure, the average stick-slip period is 41.52 s, the critical shear stress (the mean shear stress when stick-slip occurs for the first time) is 2.88 MPa, and the average stress drop is 0.64 MPa. For the A2 fault with 15 MPa of lateral pressure, the average stick-slip period is 50.63 s, the critical shear stress is 9.48 MPa, and the average stress drop is 1.31 MPa. For the A3 fault with a 25 MPa lateral pressure, the average stick-slip period is 62.32 s, the critical shear stress is 17.36 MPa, and the average stress drop is 1.83 MPa. With increasing lateral pressure, the critical shear stress, average stick-slip period and average stress drop increase. The sudden dislocation of the fault is related to the lateral pressure. When the lateral pressure is low, the fault will slip steadily. Research shows that^12^ fault stick-slip will occur only under sufficient lateral pressure. The lateral pressure affects the real contact area of asperities. The greater the lateral pressure is, the more asperities on the cross section are pressed into each other. The lateral pressure affects the interaction process of asperities and thus the sliding mode. The stress drop is the largest at high lateral pressure, and a large amount of energy is released during stick-slip motion. Many factors affect fault stick-slip instability, but none of them are independent of each other. Considering that a deep rock mass with a depth of several kilometers is in a large stress field composed of the vertical stress generated by the weight of the overlying strata and the tectonic stress generated by geological tectonic movement, the fault instability phenomenon from slow creep to stick-slip dislocation can be considered to be the result of a large amount of energy accumulation and external disturbance during excavation. Deep faults with high lateral pressure are more prone to high-magnitude fault-slip rockbursts.

## AE time-domain information

The analysis of AE characteristic parameters is performed to determine the characteristics and intrinsic law of the AE source, which is one of the most basic methods for analyzing damage to materials or structures. Based on the parametric analysis method, cumulative number of AE events, accumulation energy, magnitude, and *b* value are selected to characterize the time-domain waveform of the AE signal.

### Cumulative number of AE events

**Figure 5:**
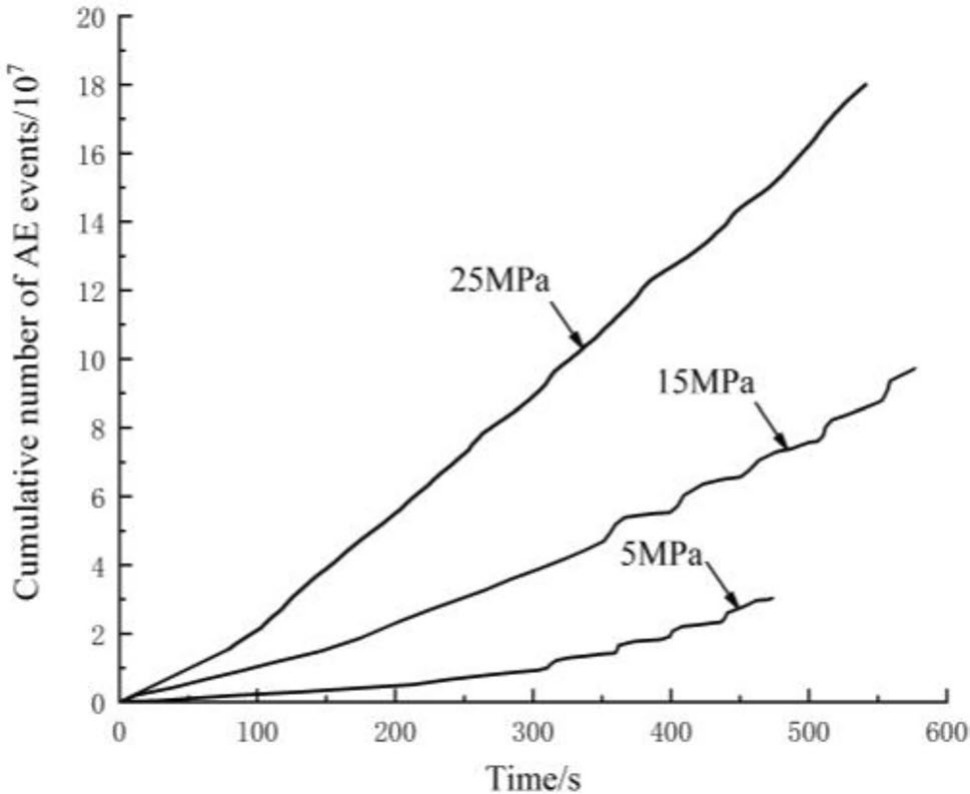
Cumulative number of AE events at different lateral pressures.

Cumulative number of AE events can reflect the total amount and frequency of AE events. Cumulative number of AE events at different lateral pressures are shown in Fig. 5.

Figure 5 shows that cumulative number of AE events increase with increasing lateral pressure, indicating that the higher the lateral pressure is, the more energy is released during stick-slip motion, and the greater the peak value of the AE count rate. When the accumulated shear stress reaches the friction strength, the asperities on the fault plane are broken, and the interactions between asperities begin to intensify. The AE events are abnormally active, and the AE count rate increases rapidly. The AE count rate increases abruptly in the meta-instability stage. The AE counts reach the maximum value at the fault slip transient. The step surge of cumulative number of AE events gradually becomes less obvious when the lateral pressure increases. This is because when the lateral pressure increases, the normal stress and shear stress increase, and the closure degree of the fault plane increases. Microfractures with asperities occur more frequently, and more latched asperities are sheared during the meta-instability stage. The fault plane slip becomes more intense, and active AE phenomena are present throughout the stick-slip process.

### Accumulation energy and magnitude

**Figure 6:**
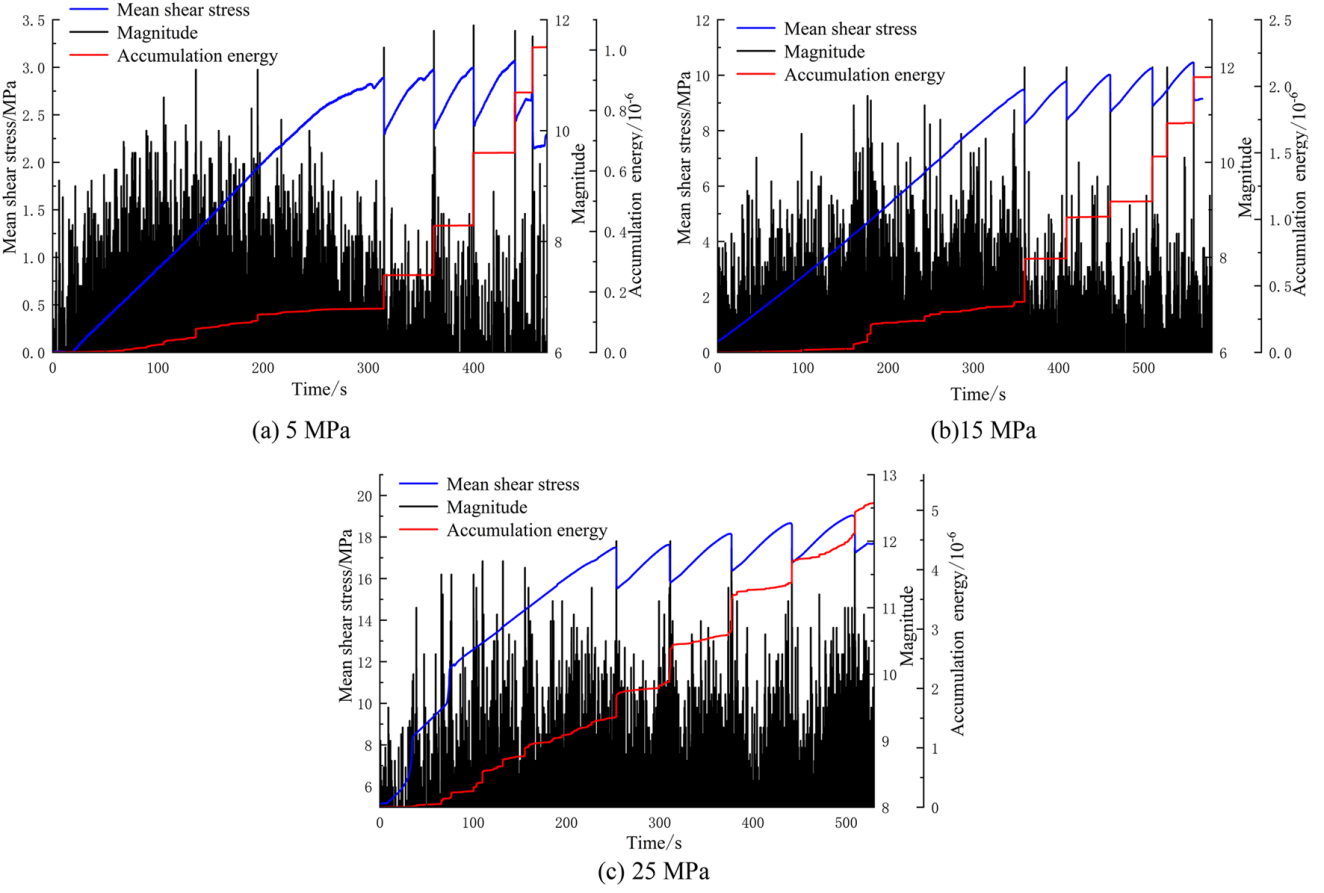
Mean shear stress-magnitude-accumulation energy evolution curves.

The accumulation energy is the cumulative value of the AE energy over time, which can reflect the energy release during rock friction. To fully display the low-energy AE events, the magnitude is used to represent the relative amplitude of the AE energy. Figure 6 shows the shear stress-magnitude-accumulation energy evolution curves during the stick-slip process of the A1–A3 faults.

In Fig. 6, the magnitude increases with increasing fault plane dislocation, revealing significant phase characteristics. In the shear stress build-up stage, the accumulation energy is stable, and the magnitude of accumulation is small. In this stage, the stress redistribution and energy release are slow, which is the quiescent episode of the AE signal. A few sparse AE energy surge points correspond to plastic deformation in localized asperities on the fault plane. The meta-instability stage is the final stage before stick-slip instability, when the stress drops sharply and high-magnitude AE events are accompanied by broken asperities. In the stick-slip instability stage, the magnitude instantaneously reaches the maximum value. There is good correspondence between the AE magnitude and the stress drop, and most of the high-magnitude signals are concentrated in the meta-instability and stick-slip instability stages. Compared with that of the A3 fault with a lateral pressure of 25 MPa, the AE quiescence episode of the A1 fault is more obvious. The amplitude of the AE energy during the quiescence episode was significantly lower than that before and after the quiescence episode. With increasing lateral pressure, the AE signal in the shear stress build-up stage gradually progresses, and the magnitude of increase intensifies. The critical shear stress, average stick-slip period, and average stress drop increase under high lateral pressure, resulting in an A3 accumulation energy that is  5 times greater than that of A1 after five slips. The above experimental results show that the accumulation energy and magnitude can characterize the friction between asperities. As the lateral pressure increases, the friction between asperities intensifies, and the number of high-magnitude AE events also increases.

**Figure 7:**
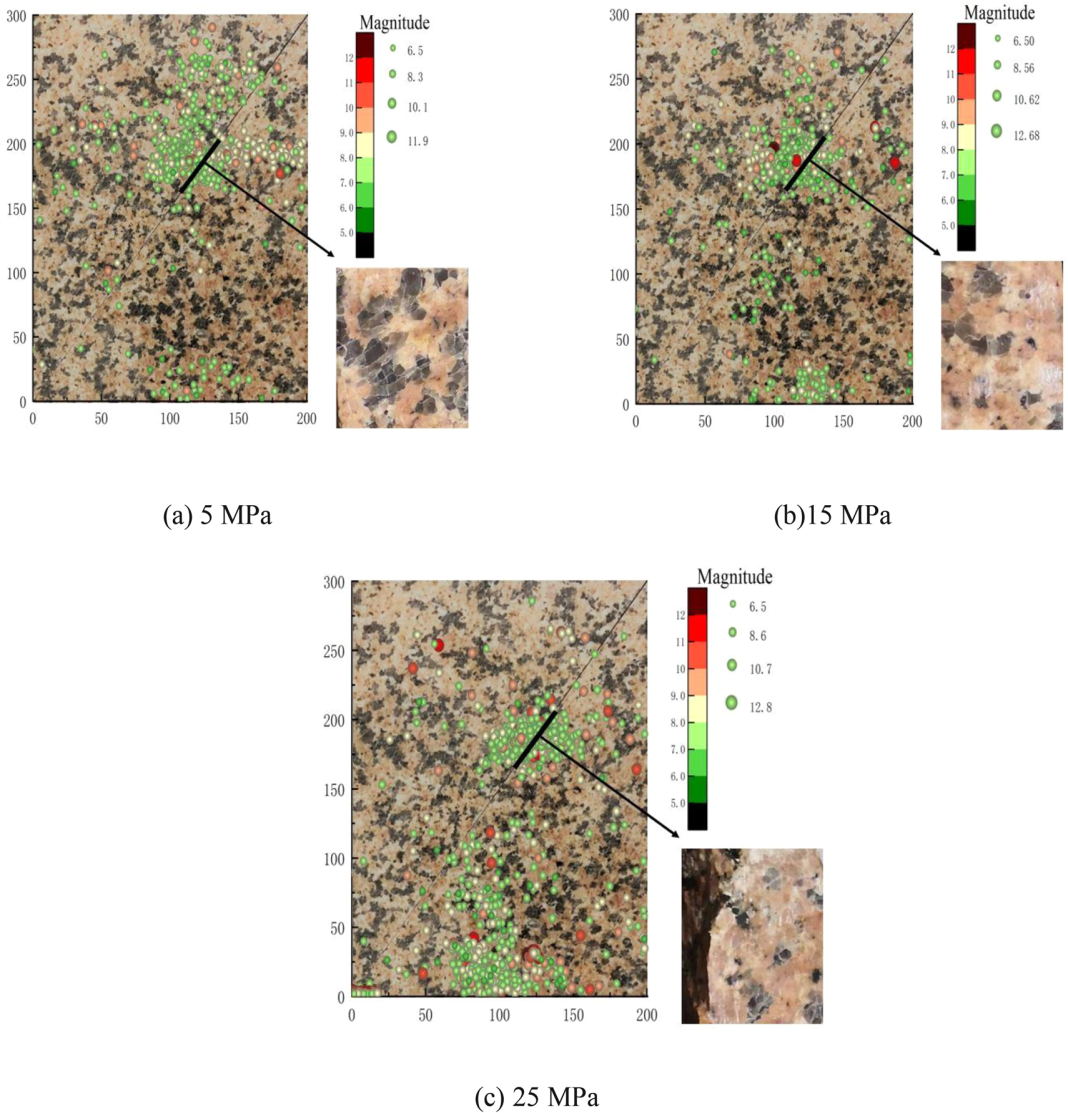
Fracture source location map and fault plane magnification map.

The intensity of AE signal depends not only on the elastic energy released by the fracture, but also on the propagation path of the elastic wave in the specimen. The attenuation and delay of the elastic wave generated by fault deformation and fracture in the propagation process cause the cause differences in the arrival time and peak value^41^, which makes the AE intensity at each channel constantly change. Based on the time difference of the AE signal from the same AE source arriving at different sensors, the location of the AE source can be roughly located. The Geiger algorithm is used to locate the AE source on the internal fracture of fault, and the results are shown in Fig. 7. The size and color of the fracture points displayed in the location map are related to the magnitude of AE event, and the higher the magnitude, the bigger the fracture points are shown, and the more the color leans towards red. As can be seen in Fig. 7, due to the existence of prefabricated fault, AE events are distributed around the faults, and their locations are relatively concentrated. White powder generated by asperities and cracks on the fault surface can be seen in the magnified fault plane. Ma et al.^42^ conducted biaxial compression of faults containing macroscopic asperities, and found that the new cracks were shear fractures across the asperities and connected to the prefabricated faults. AE events were concentrated in the pre-slip expansion area of the fault zone. The dislocations on the fault surface are not uniform dislocations, but non-uniform asperities dislocation distribution patterns^43^. The location results reflect the distribution of the actual cracks well, and the results can be considered to be accurate. Compared with 5 MPa lateral pressure, the magnitude of fracture source under 25 MPa lateral pressure is larger, which indicates that asperities are more closely occluded, and the number of asperities sheared during meta-instability stage is larger and the proportion of large-scale micro-fracture is higher under 25 MPa lateral pressure. With the increase of lateral pressure, the interaction between asperities in the cemented section and the proportion of transgranular shear fracture increase.

### b value

In rock deformation and fracture experiments, the dynamic characteristics of the acoustic emission *b* value reflect the distribution of the microfracture scale^44^. Lei et al.^45^ reported that the *b* value decreased before rock failure and instability occurred because the fracture type changed from tensile fracture to shear fracture. The crack interaction was significantly enhanced. The G-R relationships between earthquake magnitude and frequency proposed by Gutenburg and Richter are as follows^46^:

1 $$\mathit{lg}N=a-bM$$

where *M* is the magnitude, *N* is the number of events in the range of *M* + Δ*M*, and *a* and* b* are constants. The relationship between the AE magnitude and the maximum amplitude^47^ is *M* = *lgA*. Δ*M* was calculated as 0.5.

**Figure 8:**
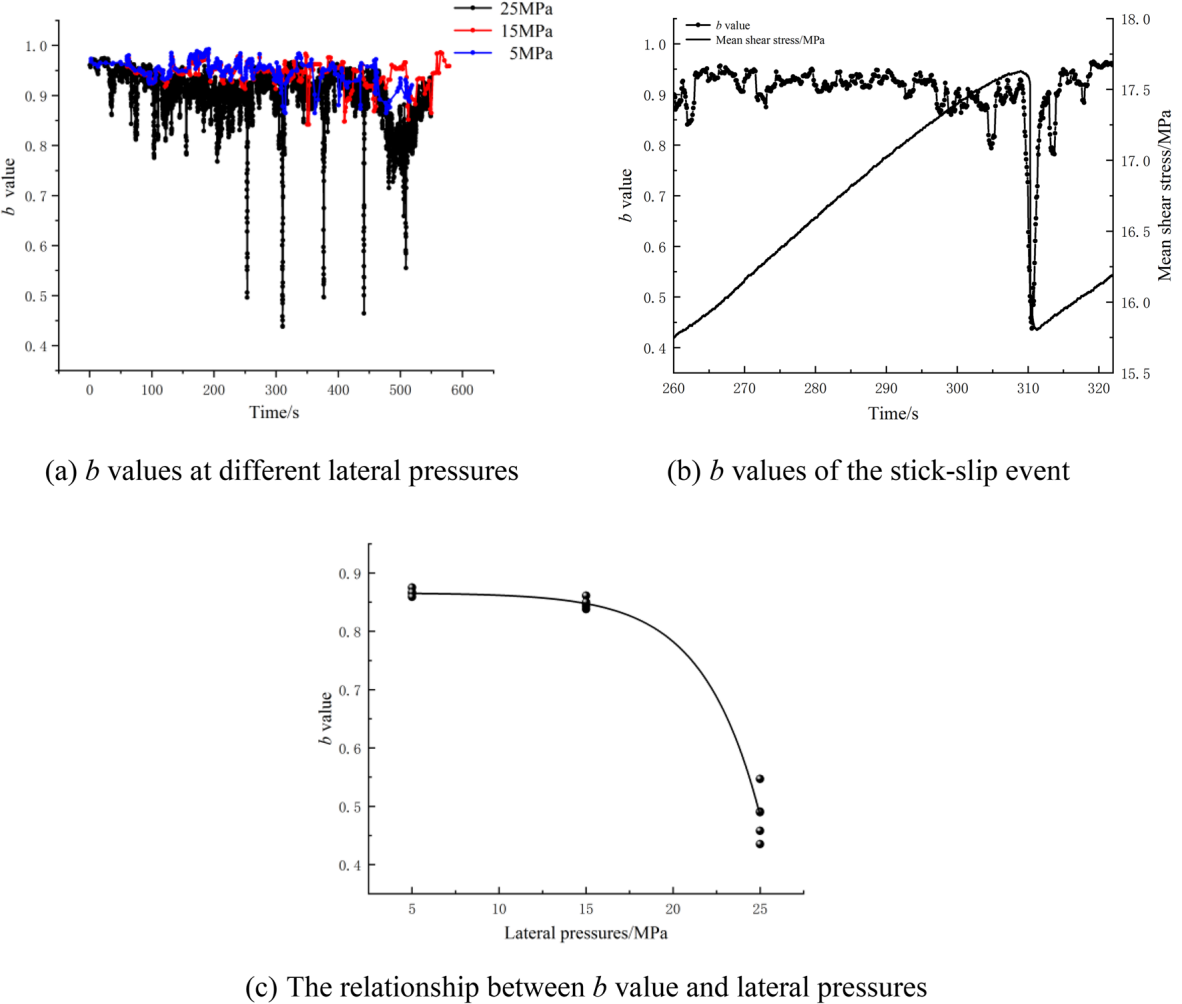
Evolution curves of the *b* value.

The linear least squares method is used to calculate the *b* value^44^, and the calculation method is as follows:

2 $$b=\frac{\sum_{i=1}^{m}{M}_{i}\sum_{i=1}^{m}lg{N}_{i}-m\sum_{i=1}^{m}{M}_{i}lg{N}_{i}}{m\sum_{i=1}^{m}{M}_{i}^{2}-{\left(\sum_{i=1}^{m}{M}_{i}\right)}^{2}}$$

where *Mi* is the i-th magnitude, and *Ni* is the count of events in the i-th magnitude. Sagar et al.^48^ reported that the sampling window and step size did not affect the *b* value evolution rule. Based on the acquisition frequency of the AE system, every 1000 AE events are set as the specimen for the *b* value calculation, and every 100 events are set as a sliding unit to prevent the calculation interval from being too small, which leads to an increase in the calculation error. Figure 8a shows the evolution curve of the *b* value during the fault stick-slip process under different lateral pressures, Fig. 8b shows the *b* value distribution of the A3 fourth stick-slip event, and Fig. 8c shows the relationship between *b* value and lateral pressures.

In Fig. 8, the *b* value evolution rules at different lateral pressures are similar; that is, the *b* value gradually decreases with stick-slip motion. In the shear stress build-up stage, the two walls of the fault are locked, the stress on the contact plane accumulates, and the internal microstructure of the fault is stable. The average *b* value is large, and the fluctuation amplitude is small, with a range of approximately 0.1. The proportion of AE events of different sizes does not change much, and the cracking status at different scales (i.e., the distribution of microfracture scales) is relatively constant. In the meta-instability stage, the fault zone connection mode changes, and the *b* value decreases continuously. It decreases to a minimum when fault instability occurs. The minimum *b* values for five stick-slip motions along the A1 fault are 0.865, 0.859, 0.875, 0.867, and 0.859. The minimum *b* values of the A2 fault are 0.838, 0.843, 0.861, 0.846 and 0.85. The minimum *b* values of the A3 fault are 0.491, 0.435, 0.49, 0.458 and 0.547, respectively. After instability occurs, the fault plane stress is adjusted, and the *b* value quickly returns to the initial level during the shear stress build-up stage. The *b* value curves of A1 and A2 have high affinity, while the *b* value curve of A3 has a high fluctuation. With increasing lateral pressure, the decrease in the *b* value during fault stick-slip instability becomes more significant, which indicates that an increase in lateral pressure can lead to an abrupt change in the fracture scale distribution of the fault plane, increase the interaction between asperities in the cemented section, and increase the proportion of transgranular shear failure. Figure 8c shows the *b* values obtained from the different stick-slip events for each lateral pressure, and the variation in the average *b* value as a function of the lateral pressure is illustrated in Fig. 8c. In addition, the fit of these data to the following formula is presented (where ${\sigma }_{X}$ is the lateral pressure and *R*^2^ is the fitting coefficient):

3 $$b = \left( {1.9E - 4} \right) \times \exp \left( {\sigma_{X} /3.3} \right) - 0.86, R^{2} = 0.983$$

**Figure 9:**
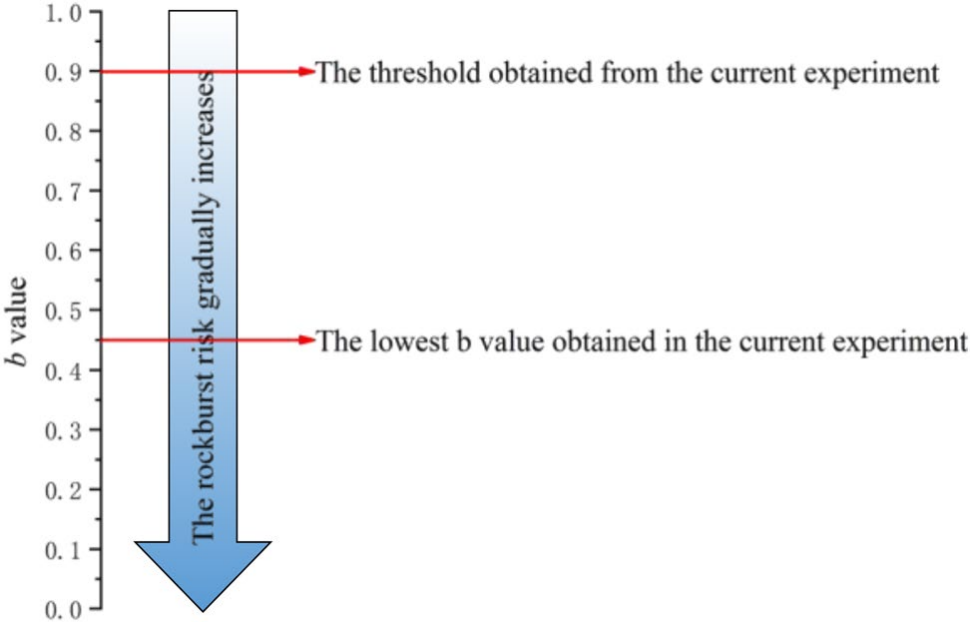
Schematic diagram of the use of the *b* value to evaluate the rockburst risk.

Above all, the *b* value tends to decrease with an increase in the lateral pressure. The minimum *b* value during the first critical shear stress lies in the range of 0.45–0.9 and it can be inferred that the *b* value may be less than 0.45 under much higher lateral pressure. Moreover, the *b* value during the shear stress build-up stage reaches approximately 0.9 when the energy surges. Based on this evidence. We can conclude that when the shear stress and energy rate are continuously increasing, the *b* value continues to decrease over time and that when the *b* value drops below 0.9, a stress drop may occur and a fault-slip rockburst may be induced. At lower *b* values, a rockburst is both more likely to occur and is likely to be more intense. Thus, an indicator is established based on the *b* value, whose threshold can be obtained through laboratory experiment. The discovery of this indicator overcomes the difficulties encountered when attempting to determine the critical AE energy which a rockburst is likely to occur. Figure 9 schematically illustrates the use of the *b* value as an indicator for evaluating rockburst risk. In Fig. 9, deeper colours correspond to higher rockburst risks. Smaller *b* values are associated with deeper colours and higher rockburst probabilities. In particular, the energy release increases significantly with increasing depth to the rockburst and reaches a minimum at the point of violent failure.

## AE Frequency spectra and multifractal characteristics

### Dominant frequency

**Figure 10:**
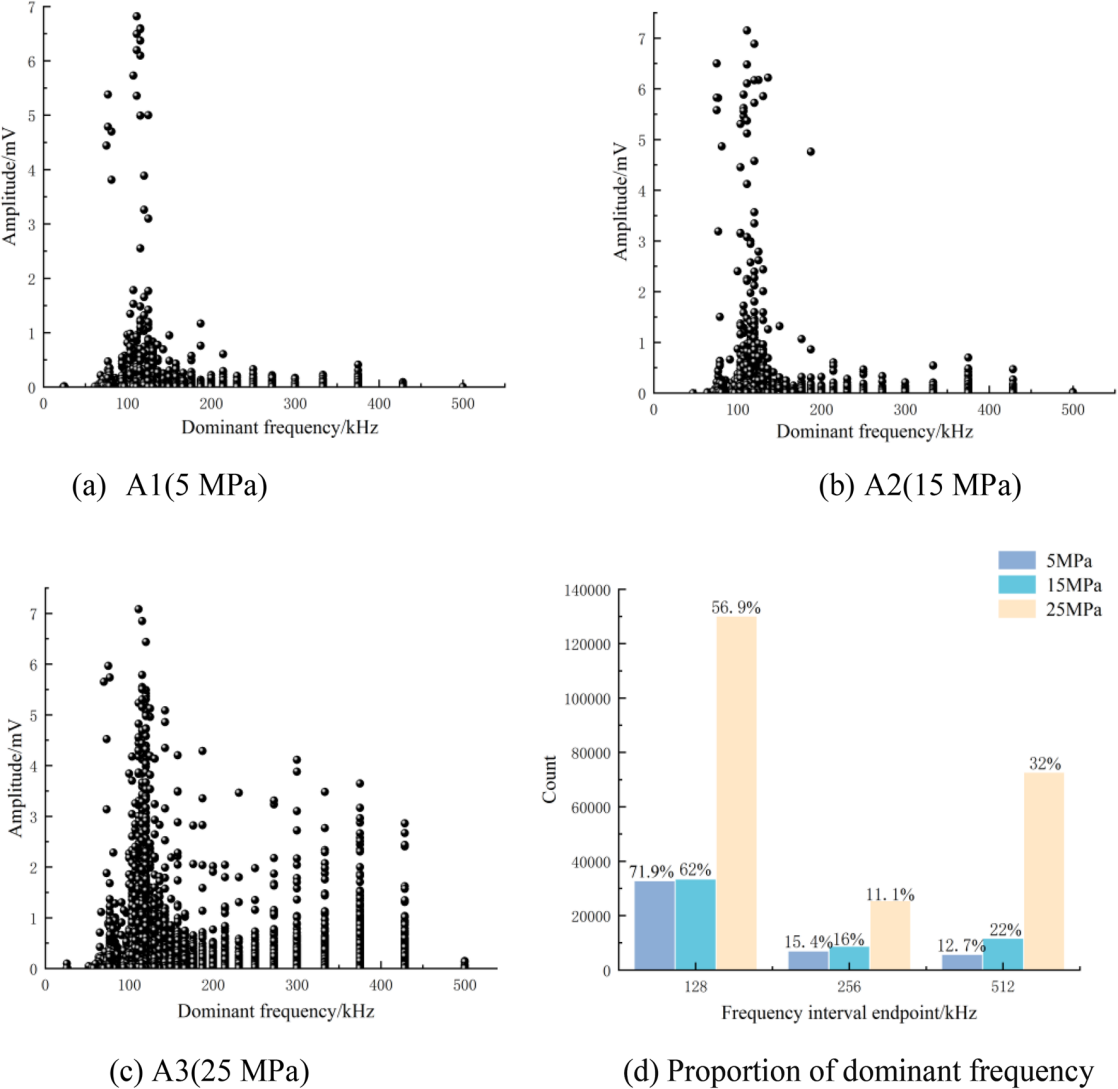
The dominant frequency distributions of AE events at different lateral pressures.

For rock, different types of seismic sources produce different scales of fractures, and different scales of fractures produce signals with different frequencies. The signals generated by large-scale cracks contain more significant low-frequency components, while the signals generated by small-scale cracks contain more significant high-frequency components. In spectrum analysis, the main frequency is an important parameter to analyze the spectrum characteristics of signals. Figure 10 shows the dominant frequency distributions of the AE signals during the fault stick-slip process at the different lateral pressures.

As shown in Fig. 10, the main frequency range of the AE signals at different lateral pressures ranges from 0 kHz to 500 kHz during the fault stick-slip process, and the overall distribution is zonal. It is mainly composed of a low-frequency band of 0 kHz–128 kHz, a middle-frequency band of 128 kHz–256 kHz and high-frequency signal points scattered in the range of 256 kHz–512 kHz. When the lateral pressure is 5 MPa, the number of AE events is low, and the dominant frequency amplitude is small. When the lateral pressure reaches 15 MPa, the number of AE events and the frequency distribution range increase. The frequency and amplitude of mid-frequency AE events increase. When the lateral pressure is 25 MPa, the dominant frequency distribution is slightly messy, the main frequency band increases, and the high-amplitude AE events in each frequency band obviously increase. The spectral distribution characteristics are slightly different under different lateral pressures. The higher the lateral pressure is, the wider the dominant frequency band distribution and the larger the amplitude. Therefore, when monitoring the AE signal of a fault stick-slip under high lateral pressure, probes with different resonant frequencies should be used to ensure that the complete AE signal can be received.

**Figure 11:**
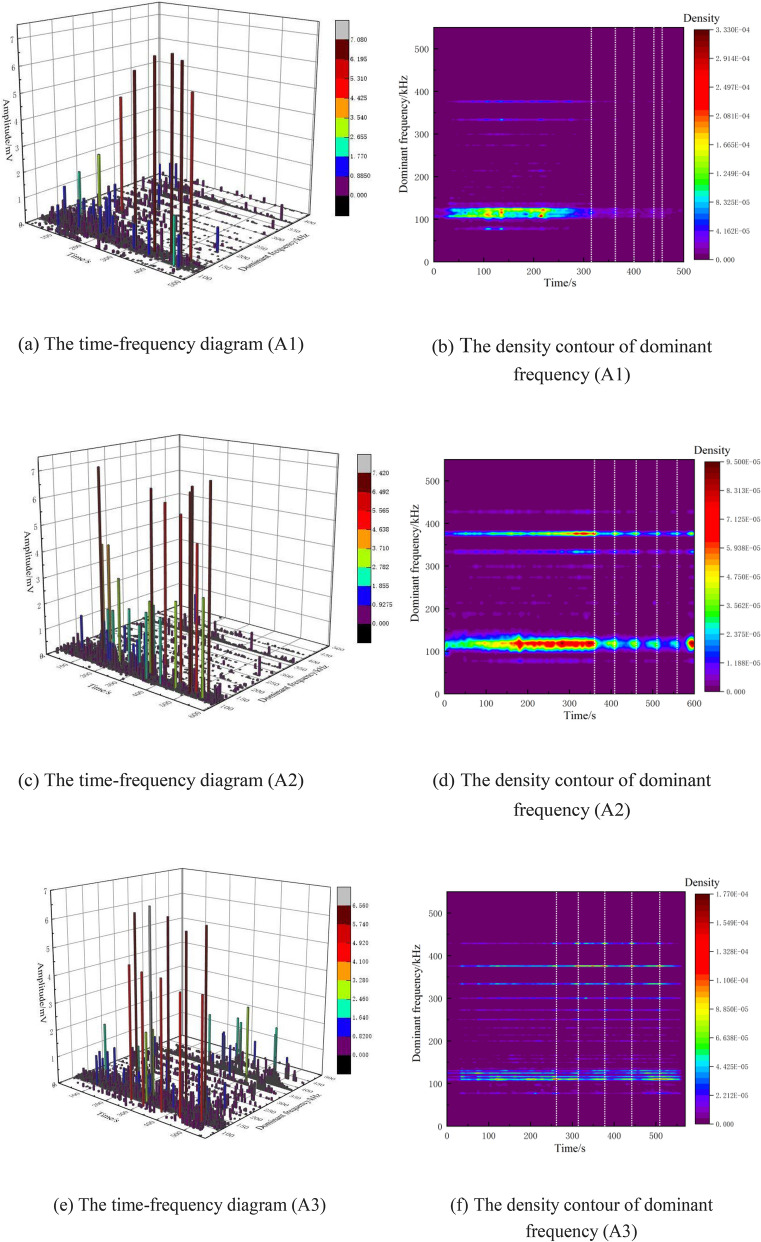
The time–frequency diagram and the density contour of dominant frequency.

According to the analysis of Fig. 11, low-frequency and low-amplitude signals mainly appear in the shear stress build-up stage, with the centre of the bands around 80 kHz and 110 kHz in the low-frequency region, and there is a blank area between 200 kHz and 300 kHz. A small number of medium- and high-frequency signals with low-amplitude appear sporadically at the same time. This indicates that in the shear stress build-up stage, there is a phenomenon of opening structure or microfissure closure inside the fault, which is manifested as small-scale fracture. In the meta-instability stage, the low-frequency and high-amplitude AE signals become more intensive than those in the previous stage, and a large number of medium- and high-frequency signals with low-amplitude appear. The frequency center is 80 kHz,120 kHz and 375 kHz respectively. AE signals in this stage are of three types: low-frequency high-amplitude, medium-frequency low-amplitude, and high-frequency low-amplitude. The large scale fracture of asperity is characterized by low dominant frequency, high amplitude and high energy, which corresponds to low-frequency and high-amplitude AE signals. The large number of frequency types implies that the fracture modes of the faults at this stage are more complicated. The amplitude of each frequency band increases due to asperities cracks penetrate and fault slip. The dominant frequency of AE signal can reflect the essential characteristics of fault stick-slip. By analyzing the dominant frequency characteristics of AE signal, the evolution law of friction and dislocation can be obtained.

### AE waveforms and frequency spectra at different loading stages

**Figure 12:**
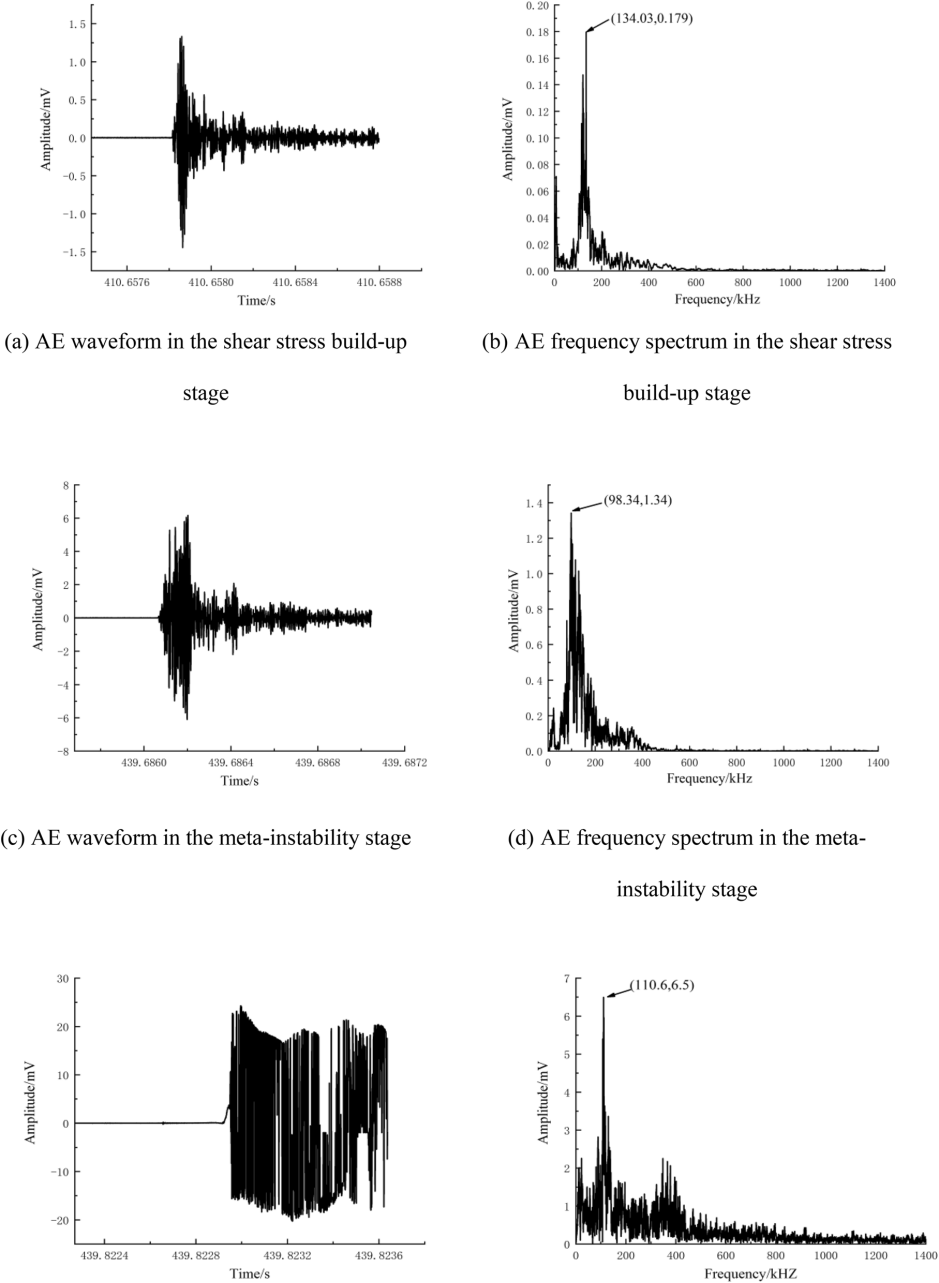
AE waveforms and frequency spectra of A1 (5 MPa).

**Figure 13:**
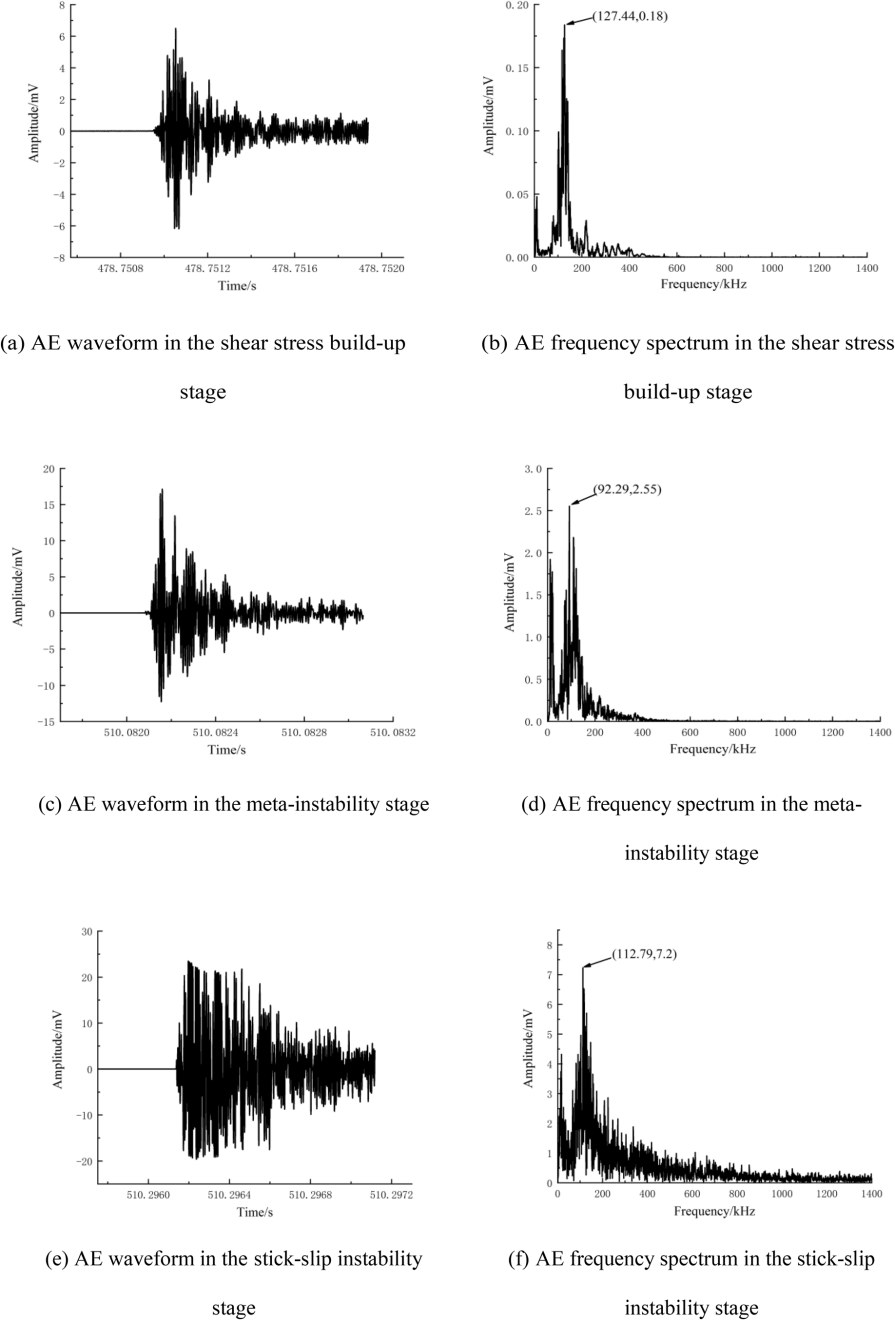
AE waveforms and frequency spectra of A2 (15 MPa).

**Figure 14:**
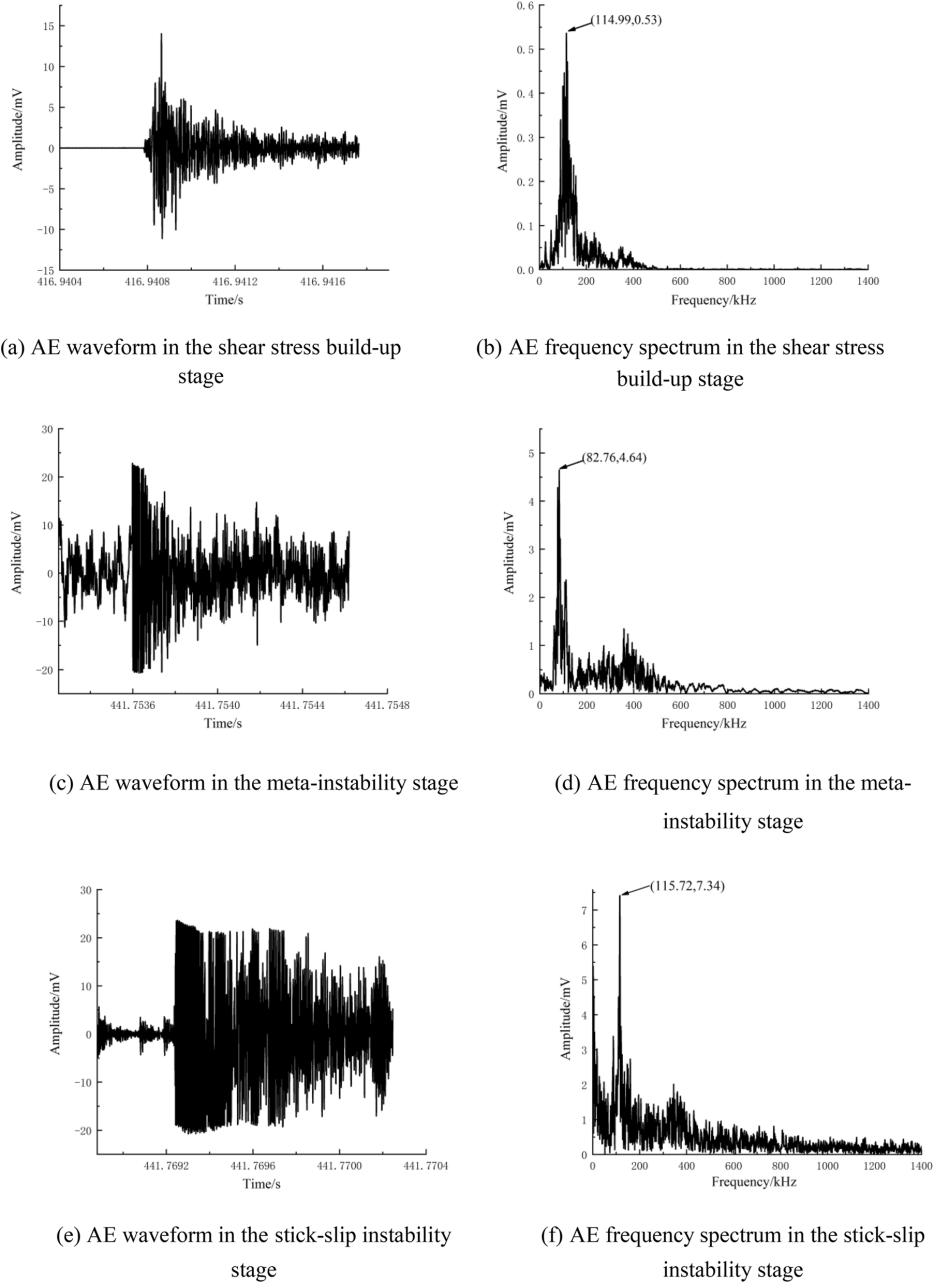
AE waveforms and frequency spectra of A3 (25 MPa).

There are two main AE sources in rocks: plastic deformation (slip deformation, etc.) and fracturing (crack formation and propagation, etc.)^49^. The AE waveform characteristics change depending on the type of AE source. Therefore, in this section, typical AE events are selected from the shear stress build-up stage, meta-instability stage, and stick-slip instability stage for analysis according to the time sequence of signal generation. The selected signals are then subjected to Fourier transform to obtain the amplitude and frequency spectrum (Figs. 12, 13 and 14). Next, refined characteristics of the waveform and frequency spectrum are studied for further revealing the AE response law of fault stick-slip.

Figures 12, 13 and 14 show that the AE waveforms generated during the fault stick-slip process are complex and nonlinear, with obvious vibration attenuation characteristics. After amplification by a 40 dB signal amplifier, the amplitude is generally in the range of 1.5–30 mV. The amplitudes of typical AE events along the A1 fault in the shear stress build-up stage, meta-instability stage, and stick-slip instability stage are 1.3 mV, 6.1 mV and 23.5 mV, respectively. The amplitudes of typical AE events in the shear stress build-up stage, meta-instability stage, and stick-slip instability stage of the A2 fault are 6.4 mV, 17.1 mV and 24.2 mV, respectively. The amplitudes of typical AE events along the A3 fault in the shear stress build-up stage, meta-instability stage, and stick-slip instability stage are 14 mv, 22.8 mv and 25 mV, respectively, with increasing tendencies. The high -amplitude AE signals are concentrated in the meta-instability stage, which can be regarded as an important period for responding to fault stick-slip motion. The number of AE events is relatively low during the stick-slip instability stage, but high amplitudes occur due to complete slip. This phenomenon also confirms the conclusion proposed by Li et al.^50^ that high-amplitude AE events are generated during the macrofailure process of rocks after reaching the peak strength. The amplitude is consistent with the change in energy release. AE waveforms at high lateral pressure have the largest local undulation and the weakest correlation between adjacent signal points. When the lateral pressure is 5 MPa, the dominant frequencies of the typical AE events in the shear stress build-up stage, meta-instability stage, and stick-slip instability stage are 134.03 kHz, 98.34 kHz and 110.6 kHz, respectively, and the amplitudes are 0.179 mV, 1.34 mV and 6.5 mV, respectively. When the lateral pressure is 15 MPa, the dominant frequencies of the typical AE events in the shear stress build-up stage, meta-instability stage, and stick-slip instability stage are 127.44 kHz, 92.29 kHz and 112.79 kHz, respectively, and the amplitudes of the three stages are 0.18 mV, 2.55 mV and 7.2 mV, respectively. When the lateral pressure is 25 MPa, the dominant frequencies of the typical AE events in the shear stress build-up stage, meta-instability stage, and stick-slip instability stage are 114.99 kHz, 82.76 kHz and 115.72 kHz, respectively, and the amplitudes of the three stages are 0.53 mV, 4.64 mV and 7.34 mV, respectively. The dominant frequencies of the three sets of experiments do not differ much, which indicates that the fracture scales are similar at different lateral pressures. An increase in lateral pressure causes fractures to occur more frequently, and the propagation speed of cracks also changes. The dominant frequency and amplitude of typical AE events increase greatly when the fault is near stick slip instability. The low-amplitude signals at the shear stress build-up stage correspond to deformations that are mainly concentrated in microdefects such as rock microfractures, voids, and pores in two walls of a fault. The low-frequency and high-amplitude signals in the meta-instability stage correspond to the fracturing of nonuniformly distributed asperities on the fault plane. The mid-frequency and high-amplitude signals in the stick-slip instability stage are related to fault dislocation. This finding is consistent with the findings of Liu^22^ and Zhu et al.^51^, who suggested that the high frequency and low amplitude and the low frequency and low amplitude signals corresponds to the inter-granular cracks or trans-crystalline micro-cracks, the low frequency and high amplitude signals corresponds to the macro-cracks, and the middle frequency and low-amplitude signals are related to the friction of existing section. Therefore, a sharp increase in the dominant frequency can be considered one of the precursory features of fault stick-slip instability.

### Multifractal characteristics of AE frequency spectra

The multifractal method can be used to describe the unstable and uneven distribution of objects. Many scholars have applied this theory to study the AE characteristics of coal and rock failure processes^26–28^. The multifractal spectrum can be calculated by the box dimension method^52^. Taking the AE frequency spectrum as an example, the frequency sequence is recorded as {*x*_*i*_}, and {*x*_*i*_} is divided into *N* subsets of length *n*. If the sequence {*x*_*i*_} satisfies the multifractal feature, at *n → 0*, the probability distribution functions {*P*_*i*_(*n*)} and *n* of its subset satisfy the following formula^52^:

4 $$\{{P}_{i}(n)\}\propto {n}^{\alpha }$$

where *α* is the singularity constant, reflecting the degree of inhomogeneity of the {*x*_*i*_} probability subset.

If the subset marked by *α* has the same probability, the number of units is recorded as *N*_*α*_(*n*)*.* In general, the smaller the division scale *n* is, the more the subsets are obtained; thus, *N*_*α*_(*n*) increases with decreasing *n*, and they have the following relationship:

5 $${N\alpha (n)\propto n}^{-f\left(\alpha \right)}$$

where *f*(*α*) is the fractal dimension of the subset represented by* α*, indicating the frequency of the subset in the whole subset set.

First, a partition function is defined^52^:

6 $${X}_{q}\left(n\right)=\Sigma {P}_{i}{\left(n\right)}^{q}={n}^{\tau \left(q\right)}$$

where $\tau \left(q\right)$ is the quality index; *q* is the weight factor, which represents the proportion of probability density *P*_*i*_(*n*) of different sizes in the partition function ${X}_{q}\left(n\right)$, and its value can be changed. Therefore, the value of *q* can be limited to a certain range in the actual calculation.

The value of *τ*(*q*) can be calculated by the slope of the logarithmic curve ${\text{ln}}{X}_{q}\left(n\right)-{\text{ln}}n$:

7 $$\tau (q)=\underset{n\to 0}{lim}\frac{{\text{ln}}{X}_{q}\left(n\right)}{{\text{ln}}n}$$

The *q*-order multiple fractal dimension *D*_*q*_ of sequence {*xi*} can be solved by the following formula:

8 $$Dq=\left\{\begin{array}{c}\frac{1}{q-1}\underset{n\to 0}{lim}\frac{{\text{ln}}\Sigma {P}_{i}{\left(n\right)}^{q}}{{\text{ln}}n}\left(q\ne 1\right)\\ \underset{n\to 0}{lim}\frac{\Sigma {P}_{i}{\left(n\right)}^{q}{\text{ln}}{P}_{i}{\left(n\right)}^{q}}{{\text{ln}}n}\left(q=1\right)\end{array}\right.$$

The curve composed of the multiple fractal dimensions *D*_*q*_ and the weighting factor *q* is the generalized fractal curve of the sequence {*xi*}. The larger “*D*_*q*_ − 1” is, the greater the volatility of the data and the stronger the multifractal feature.

Applying the Legendre transformation to *τ*(*q*)*-q*, the singularity constant *α* and the fractal dimension *f*(*α*) of the represented subset can be calculated by the following formulas^52^:

9 $$\alpha =\frac{d\left(\tau (q)\right)}{dq}=\frac{d}{dq}\left(\underset{n\to 0}{lim}\frac{{\text{ln}}{X}_{q}\left(n\right)}{{\text{ln}}n}\right)$$

10 $$f\left(\alpha \right)=\alpha q-\tau (q)$$

**Figure 15:**
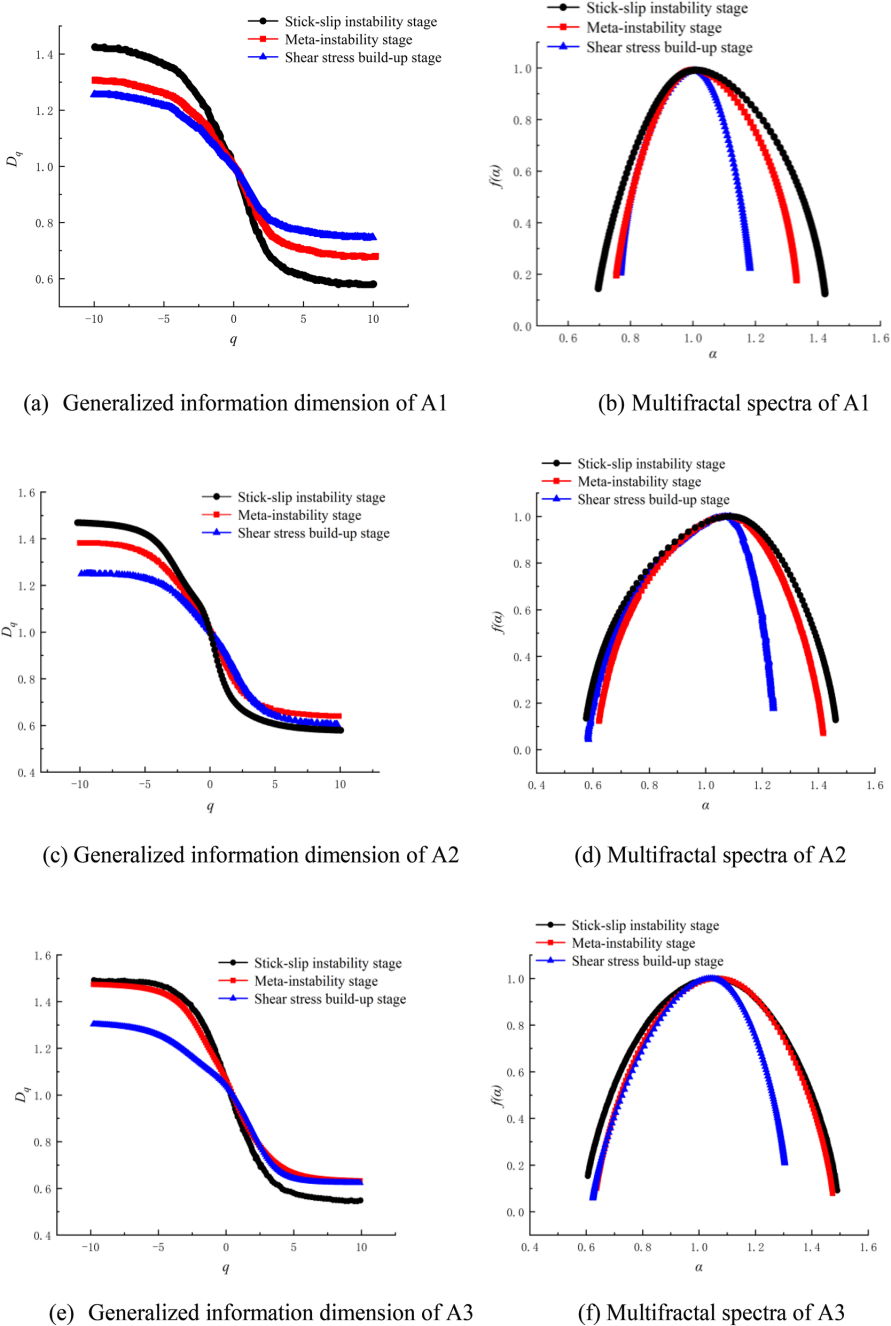
Generalized information dimension and multifractal spectra of AE frequency spectra.

According to formulas (8), (9) and (10), the multifractal characteristic parameters of the AE frequency spectra during the fault stick-slip process under different lateral pressures were calculated. The generalized information dimension and multifractal spectra of the AE frequency spectra in Figs. 12, 13 and 14 were calculated via MATLAB software, as shown in Fig. 15.

The multifractal spectrum is a curve formed by *α* and *f*(*α*), which can reflect the uneven distribution characteristic of the frequency amplitude. For the multifractal spectrum, the subset represented by *α*_*min*_ corresponds to small amplitude frequencies, the subset represented by *α*_*max*_ corresponds to large amplitude frequencies, and the width of the multifractal spectrum Δ*α* = *α*_*max*_* − α*_*min*_ describes the unevenness of the frequency amplitude. The larger the spectral width Δ*α* is, the greater the difference in the frequency distribution and the more severe the fluctuation. Let Δ*f*(*α*) = *f*(*α*_*max*_)* − f*(*α*_*min*_); then, Δ*f* represents the ratio of the frequency amplitude in the relevant sequence subset at the maximum and minimum. A smaller Δ*f*(*α*) represents a greater proportion of large -amplitude frequencies in the sequence, and vice versa.

Figure 15 suggests that the multifractal dimensions *D*_*q*_ of frequency spectra of the three groups of AE signals are monotonic decreasing functions of *q*. This means that the frequency distributions of the AE signals have multifractal characteristics and are not uniformly distributed. The value of the multifractal dimension *D*_*qmax*_ corresponding to the minimum value of the weight factor *q*_*min*_ can reflect nonuniformity and multifractal characteristics. The larger the *D*_*qmax*_ is, the more notable the multifractal characteristics are. The shapes of the multifractal spectra at different lateral pressures are similar. The *f*(*α*)*-α* spectra are asymmetric at both ends. The *f*(*α*_*min*_) end of AE frequency spectra in the meta-instability stage, and stick-slip instability stage are relatively high, and Δ*f* are all smaller than 0. This means that large wave peaks in AE frequency spectra account for large proportions. The widths of the multifractal spectra (Δ*α*) vary significantly at different stages, which implies that the distribution of the AE spectra varies significantly among the various stages of fault stick-slip. At a lateral pressure of 5 MPa, the maximum multifractal dimensions (*D*_*qmax*_) of the typical AE spectra in the shear stress build-up stage, meta-instability stage, and stick-slip instability stage are 1.24, 1.345 and 1.469, respectively, and the widths of the multifractal spectra (Δ*α*) are 0.406, 0.576 and 0.732, respectively. At a lateral pressure of 15 MPa, the maximum multifractal dimensions (*D*_*qmax*_) of the typical AE spectra in the shear stress build-up stage, meta-instability stage, and stick-slip instability stage are 1.237, 1.375 and 1.461, respectively, and the widths of the multifractal spectra (Δ*α*) are 0.656, 0.807 and 0.88, respectively. At a lateral pressure of 25 MPa, the maximum multifractal dimensions (*D*_*qmax*_) of the typical AE spectra in the shear stress build-up stage, meta-instability stage, and stick-slip instability stage are 1.303, 1.473 and 1.491, respectively, and the widths of the multifractal spectra (Δ*α*) are 0.679, 0.841 and 0.943, respectively. The *D*_*qmax*_ and Δ*α* of the typical AE spectrum increase during the fault stick-slip process. In the meta-instability stage, the AE amplitude fluctuates sharply with frequency, the peak value of the dominant frequency is large, and the ratio of nonpeak-value frequencies to peak-value frequencies is reduced. Thus, these nonpeak-value frequencies become relatively weak frequencies, which leads to increases in *D*_*qmax*_ and Δ*α*. The higher the lateral pressure is, the greater the *D*_*qmax*_ and Δ*α* are. An increase in *D*_*qmax*_ and Δ*α* indicates that the multifractal characteristics of the AE spectrum are gradually enhanced, and the difference between the dominant and nondominant frequencies gradually increases, which leads to increased inhomogeneity. This might be because as the lateral pressure increases, the fracture asperities increase, and the number of cracks increases correspondingly^53^, leading to a gradual increase in and concentration of dominant frequency amplitude points. The multifractal parameters of the AE spectrum differ greatly under different lateral pressures, but the dynamic variations in the multifractal parameters are similar. This suggests that the microscopic complexity of AE events generated by faults under different lateral pressures varies, but the macroscopic generation mechanism of AE events has inherent uniformity. Further research is expected to identify the stage of fault stick-slip motion through the frequency of AE signals, which is meaningful for the study of rock mechanics and seismology. Using multifractal theory and methods to study the characteristics of the AE frequency spectrum is helpful for identifying the characteristics of precursor signals of fault stick-slip instability.

## Discuss

The asperities and weak segments within the fault plane impact the dynamic fracture propagation process. During the loading process, asperities generate localized stress concentrations, thus forming the fracture nucleation region. The continuous accumulation of damage causes the nucleation regions to expand and gradually penetrate to form a macroscopic fracture plane, which ultimately leads to the accelerated release of shear strain. The fault stick-slip motion shows the destruction of many force chain networks between asperities on the microscopic level. When the stress exceeds the strength of the force chain, the force chain breaks and releases the strain energy at the same time. Part of the strain energy propagates outward in the form of an elastic wave, generating the AE signal. In the process of force chain fracture, if the load work is *U*, the increased energy in the asperity system is transformed into the released elastic strain energy *U*^*e*^ and dissipated strain energy *U*^*d*^. From the law of energy conversion:

11 $$U={U}^{e}+{U}^{d}$$

In the bidirectional shear friction experiment, the input strain energy $Us$ is:

12 $${U}_{s}={\int }_{0}^{{\varepsilon }_{i}}{\sigma }_{\dot{i}}d{\varepsilon }_{i}+{\int }_{0}^{{\varepsilon }_{j}}{\tau }_{j}d{\varepsilon }_{j}$$

where ${\sigma }_{\dot{i}}$ and ${\tau }_{j}$ are the normal stress and shear stress applied to asperities, respectively; and ${\varepsilon }_{i}$ and ${\varepsilon }_{j}$ are the positive strain and shear strain, respectively.

The input elastic strain energy ${U}_{s}^{e}$ is:

13 $${U}_{s}^{e}=\frac{1}{2}{\sigma }_{\dot{i}}{\varepsilon }_{i}+\frac{1}{2}{\tau }_{j}{\varepsilon }_{j}$$

From generalized Hooke's law:

14 $${\varepsilon }_{i}=\frac{\left({\sigma }_{\dot{i}}-v{\tau }_{j}\right)}{2E}$$

15 $${\varepsilon }_{j}=\frac{\left({\tau }_{j}-v{\sigma }_{\dot{i}}\right)}{2E}$$

where $E$ is the elastic modulus and $v$ is Poisson's ratio.

The ring-down count of the AE is

16 $$\eta =\frac{f}{\beta }\mathit{ln}\frac{{V}_{P}}{{V}_{t}}$$

where ${V}_{t}$ is the threshold voltage; ${V}_{P}$ is the peak voltage; $\beta$ is the attenuation coefficient; and $f$ is the signal frequency, which is roughly equal to the resonant frequency of the sensor.

The relationship between ${V}_{P}$ of the signal received by the sensor and the elastic strain energy ${U}^{e}$ is as follows:

17 $${V}_{P}=\psi \sqrt{{U}^{e}}$$

where $\psi$ is the electromechanical conversion constant.

The attenuation coefficient $\beta$ is related to the frequency $f$, whereas the electromechanical conversion constant $\psi$, threshold voltage ${V}_{t}$ and energy ${U}^{e}$ are determined by the AE acquisition system and the test itself. In Eq. (16), the ring-down count $\eta$ is closely related to the frequency $f$. The calculation formula of the discrete Fourier transform is:

18 $$X\left(k\right)=\sum_{n=0}^{N-1}x\left(n\right){W}_{N}^{nk}$$

19 $${W}_{N}={e}^{-j\frac{2\pi }{N}}$$

where $x\left(n\right)$ is the original digital signal sequence in the time domain.

The amplitude of the AE power spectrum is:

20 $$X\left(k\right)=\sum_{n=0}^{N-1}\frac{f}{\beta }\mathit{ln}\frac{\psi \sqrt{{\sigma }_{i}^{2}+{\tau }_{j}^{2}-2v{\tau }_{j}{\sigma }_{\dot{i}}}}{{V}_{t}\sqrt{2E}}{e}^{-j\frac{2nk\pi }{N}}$$

During the loading process, part of the elastic strain energy ${U}^{e}$ stored in the fault propagates outwardly in the form of an AE signal, and the amplitude of the AE power spectrum is related to the stress and elastic strain energy released by asperities. The increasing stress constantly breaks the equilibrium state of the force chain system between asperities. Fracture and reconstruction of the force chain system cause asperity dislocation. As the lateral pressure increases, the number of fractured asperities increases. The elastic strain energy ${U}^{e}$ is released, which manifests as enhanced AE activity and increased AE intensity.

The difference in AE intensity is caused by the slip and reconstruction of asperities in different stages. A large number of asperities fracture during the meta-instability stage. The expansion of the fracture nucleation region is fastest near the peak stress, resulting in a large release of elastic strain energy ${U}_{s}^{e}$. As shown in Eq. (20), an extremely strong AE signal is generated at this time, and the amplitude of the power spectrum increases. The evolution rate of acoustic emission responses accelerates when fault stick-slip instability approaches^54^, and the *b* value of acoustic emission events abnormally decreases before instability^21^. This is consistent with the inverse Omori law of foreshock activity in natural earthquakes^55^. Several scholars have proposed different explanations for this phenomenon. For example, Latour et al.^56^ and Kaneko et al.^57^ studied the extension of the fault rupture length through experiments and numerical simulations, and concluded that the phenomenon of an increase in the power law near instability is related to the acceleration process of fault rupture. Wang et al.^58^ derived the distribution function solutions of displacement and energy fields characterizing fault sliding, built a physical model to simulate fault sliding, and proposed that the mutation effect of fault and overburden rock strata instability was related to the drastic change in the stress on the fault surface, the accelerated change in the fault displacement and the large and frequent fluctuation in the strain energy. Zhuo et al.^59^ reported that an accelerated increase in the cumulative fault displacement may result in critical acceleration of responses when the local preslip area transforms from a state of quasistatic extension to quasidynamic extension. Helmstetter et al.^60^ believe that the ETAS model can explain the power-law surge phenomenon of meta-instability. In the stick-slip instability stage, a variety of fine particles appear in the macroscopic fracture zone due to bulk deformation and structural deformation. With the friction between particles, the elastic strain energy accumulated in the fault is released, and the dominant frequency of AE is scattered. The frequency is inversely proportional to the contact time of the force chain^61^, and the contact time is proportional to the final displacement of the asperities; therefore, the AE frequency is inversely proportional to the final displacement of the asperities. With the imbalance of the force chain system during the meta-instability stage, the final displacement increases, and the corresponding AE frequency decreases.

Based on the above analysis, the AE frequency with phased response characteristics can be used to effectively determine the evolution of fault stick-slip instability at the laboratory scale. The dominant frequency and fractal parameters of AE are suitable as the main parameters for the prediction of dynamic disasters such as rockburst. The dominant frequency can reflect the intrinsic characteristics of fault and is a sensitive AE parameter for determining the rock structure and stress state. The fractal parameters of the AE spectrum can reflect the overall response law. In coal mine monitoring, fractal parameters can be programmed into the monitoring system. When the monitored fractal parameters change abruptly, it can be assumed that there are a large number of macroscopic fracture zones on the fault. In practical applications, it is necessary to determine the occurrence environment, structure and stress characteristics of rock masses in monitoring areas through field investigations and preliminary basic research. On this basis, appropriate AE parameters should be selected to obtain more accurate precursor information, and a multiparameter combined forecasting system should be established to improve the accuracy of coal and rock dynamic disaster prediction.

## Summary

In this paper, the macroscopic statistical parameters (cumulative number of AE events, magnitude and *b* value) and local characteristic parameters (amplitude and dominant frequency) during the process of fault stick-slip under different lateral pressures are investigated. The nonlinear characteristics of AE waveforms spectra are analyzed with the multifractal theory. On this basis, the microscopic mechanism of fault stick-slip is discussed. The main conclusions are as follows:

1. Changing the lateral pressure in the bidirectional shear friction experiment results in a change in the stress state of the fault plane, which subsequently changes the friction strength of the fault and stick-slip characteristics. The critical shear stress, average stress drop and average stick-slip period increase when the lateral pressure increases.
2. With the increase of lateral pressure, the interaction between asperities in the locking section of the fault plane is enhanced, and the proportion of transgranular shear fracture is increased, which leads to an increase in the cumulative number of AE events and magnitude. Most high-magnitude AE events are concentrated in the meta-instability stage and stick-slip instability stage. There is a good correlation between a high-magnitude AE event and a stress drop, rather than between high-magnitude AE events and stress. The periodic decrease in the *b* value is more significant at high lateral pressure. When the *b* value drops below 0.9, the stress may decrease and the rock burst may be induced. At lower *b* values, rockbursts are more likely and more intense.
3. The AE spectral distribution characteristics at different lateral pressures are slightly different. The higher the lateral pressure is, the wider the distribution of the dominant frequency band and the larger the amplitude. The AE frequency with phased response characteristics can be used to effectively determine the evolution of fault stick-slip instability at the laboratory scale, anda sharp increase in the amplitude of the dominant frequency can be regarded as one of the precursory features of fault stick-slip instability.
4. At different lateral pressures, the multifractal spectral shapes of the AE frequency spectra and dynamic trends of the fractal parameters are similar, but the values of the fractal parameters are quite different. This indicates that the microscopic complexities of AE events generated by faults under different lateral pressures differ, but the macroscopic generation mechanisms of AE events are intrinsically consistent. The larger the fracture scale of asperities is, the more significant the multifractal characteristic of the AE frequency spectra. The maximum multifractal dimension (*D*_*qmax*_) and spectral width (Δ*α*) can reflect the difference in energy released during fault stick-slip motion.

## Data Availability

The datasets generated and/or analyzed during the current study are not publicly available due [REASON WHY DATA ARE NOT PUBLIC] but are available from the corresponding author on reasonable request.
